# PSMC2 is overexpressed in glioma and promotes proliferation and anti-apoptosis of glioma cells

**DOI:** 10.1186/s12957-022-02533-1

**Published:** 2022-03-14

**Authors:** Xiaoyang Zheng, Yuguang Wang, Dongxu Wang, Jingru Wan, Xiangying Qin, Zhuang Mu, Nan Hu

**Affiliations:** 1 Imaging Center, The second Affiliated Hospital, Qiqihar Medical University, No. 37 Zhonghua West Road, Jianhua District, Qiqihar, 161000 Heilongjiang Province China; 2 Department of Brain Surgery, The second Affiliated Hospital, Qiqihar Medical University, Qiqihar, 161000 China; 3 Department of Brain Surgery, the First Hospital of Qiqihar City, Qiqihar, 161000 China; 4 Center of Pathology, Qiqihar Medical University, Qiqihar, 161000 China

**Keywords:** PSMC2, Glioma, Apoptosis, Survival analysis

## Abstract

**Background:**

This study aims to investigate the effect of PSMC2 expression on the clinical prognosis of glioma patients and its molecular mechanism.

**Methods:**

TCGA multi-tumor screening and survival analysis were combined to explore the differential expression of PSMC2 in multi-tumor. PSMC2 expression in glioma and normal tissues was detected by Western blot and RT-qPCR. Kaplan-Meier survival curve was used to visualize the effect of PSMC2 expression on the overall survival rate and disease-free survival rate of patients with glioma. The highly expressed cell line U343MG was selected to construct a PSMC2 knockdown model by siRNA transfection, and the effect of PSMC2 knockdown on cell proliferation ability was evaluated by CCK-8 assay. Gene-set enrichment analysis of PSMC2 co-expression genes was carried out to predict the molecular mechanism of their regulation of tumor cell phenotypes, and the analysis results were verified by flow cytometry and Western blot.

**Results:**

Through broad-spectrum screening of 31 kinds of tumors, we found that PSMC2 was upregulated in most tumors, but PSMC2 was most significantly overexpressed in gliomas and correlated with poor prognosis in glioma patients. The results of Western blot and qRT-PCR showed that PSMC2 was significantly overexpressed in glioma tissues. Further survival analysis revealed that the overall survival and disease-free survival of patients with low PSMC2 expression were significantly better than that of patients with high PSMC2 expression. The proliferation of U343MG cells was significantly inhibited after PSMC2 knockdown. Enrichment analysis of PSMC2 co-expression genes indicated that PSMC2 affected the apoptosis process. The expression of apoptosis-related proteins also significantly changed following PSMC2 knockdown.

**Conclusions:**

PSMC2 promotes the proliferation of glioma cells and inhibits the apoptosis, which is expected to be a potential therapeutic target for glioma.

**Supplementary Information:**

The online version contains supplementary material available at 10.1186/s12957-022-02533-1.

## Introduction

Glioma is one of the most common primary brain tumors, accounting for 60% of all brain tumors and presenting with varying degrees of malignancy. It is particularly challenging to treat the disease because of the poor prognosis and limited treatment options [1]. Despite advances in surgery and adjuvant therapy, brain tumors remain one of the leading causes of cancer-related mortality and morbidity in adults and children [2, 3]. Among them, patients with glioblastoma, the most aggressive type of glioma, have a median survival time of only 14–18 months due to rapid tumor growth and resistance to current therapies and drugs [4, 5]. Therefore, it is pressing to find potential new targets and effective drugs to improve the prognosis of patients with glioma.

It is critical to maintain the integrity of proteome for all cells, especially tumor cells. The ubiquitin-proteasome system (UPS) is an important component to maintain protein homeostasis. The 26S proteasome, consisting of a 20S core catalytic subunit and a 19S regulatory subunit, is responsible for protein degradation in eukaryotic cells. The target protein substrate with polyubiquitin labeling is first recognized by the 19S regulatory subunit and then expanded and transported to the 20S core subunit for degradation [6]. One or two 19S regulatory complexes can combine with a single 20S catalytic complex to form a single-ended or double-ended (26S1 or 26S2) intact 26S proteasome [7]. By specifically clearing target proteins, 26S proteasomes are involved in almost all biological activities, such as cell division, angiogenesis, immune response, transcription factor activation, and posttranslational modification of proteins [8, 9]. Given the importance of 26S proteasome, it is a multifaceted target for anticancer therapy [10]. In fact, targeting 26S proteasomes has been proved to be successful in the treatment of invasive hematopoietic tumors [11, 12].

Proteasome 26S subunit ATPase 2 (PSMC2), a newly identified gene located on chromosome 7q21.1q22.3, encodes a protein that is an important member of the 19S proteasome [7, 8]. It is the key to the assembly of 19S and 26S proteasomes [13]. In a study on genomic instability in cancer published in *Cell* in 2012, Nijhawan et al. found a partial deletion of PSMC2, which makes cancer cells highly dependent on the remaining PSMC2, suggesting that PSMC2 could be a potential target for cancer treatment [14]. There are other studies showing that PSMC2 plays an important role in a number of cancers, such as osteosarcoma [13], pancreatic cancer [15], and human alveolar soft tissue sarcoma [16].

To date, however, there are no reports about the expression of PSMC2 in broad-spectrum tumors and its effect on the survival of patients, especially on gliomas. Therefore, this study aims to explore the effect of PSMC2 expression on glioma and its molecular mechanism. Through the RNA-Seq data of TCGA (The Cancer Genome Atlas) and GTEX (Genotype-Tissue Expression), we found that PSMC2 was significantly upregulated in glioma and was associated with patient survival. PSMC2 knockdown assay and apoptosis assay showed that PSMC2 knockdown inhibited cell proliferation and promoted cell apoptosis. Thus, PSMC2 may be a potential target for the treatment of glioma.

## Materials and methods

### Data sources

This study made use of data from public databases. RNA-Seq data of glioma patients and corresponding clinicopathological data were obtained from TCGA (The Cancer Genome Atlas) and GTEX (Genotype-Tissue Expression) databases. In addition, dataset GSE53733 of GeneChip Transcriptome Array was obtained from Gene Expression Omnibus (GEO) database (http://www.ncbi. nlm.nih.gov/geo/).

### Clinical sample information

Thirty cases of glioma tissues and negative control samples were included in this study, which were obtained from neurosurgical resection in our hospital from November 2018 to November 2019. None of the patients had received adjuvant therapy such as radiotherapy, chemotherapy, or immunotherapy before surgery. Two pathologists independently diagnosed the histopathological features of glioma tissues. There were 30 cases of negative control tissues, which were normal brain tissues from patients with craniocerebral trauma who were admitted to our hospital during the same period. All tissue specimens were stored at −80 °C for later use. Patients and their families involved in this study voluntarily participated and signed the informed consent. This study was approved by the Medical Ethics Committee of our hospital.

### Cell culture

Glioma cell lines AM38, A172, LN229, U118MG, U87MG, U251MG, and U343MG were all obtained from China Center for Type Culture Collection (CCTCC, Shanghai, China). A complete medium was prepared by adding 10% fetal bovine serum (FBS; Gibco, USA), 1% penicillin, and 1% streptomycin (Invitrogen, USA) in Dulbecco’s Modified Eagle’s medium (DMEM; HyClone, USA). The cells were cultured in complete medium in a constant temperature incubator at 37 °C, 5% CO_2_, and saturated humidity. The complete culture medium was changed every 48 h. Subculture was carried out when the cell confluence reached 90%, and subsequent cell experiment was conducted after the stable passage to the 3rd or 4th generation.

### Small interfering RNA transfection

According to the experimental requirements, cells were divided into three groups: siNC (negative control), si PSMC2-1, and si PSMC2-2. The target sequence of PSMC2 siRNA is si PSMC2-1: 5′-GCCAGGGAGATTGGATAGAAA-3′; si PSMC2-2: 5′-GGTGGCACCTACTGACATTGA-3′; and the target sequence of negative control siNC is 5′-GCACTACCAGAGCTAACTCAGATAGTACT-3′ (GenePharma, Shanghai, China). The day before the transfection experiment, the cell lines in logarithmic growth phase were inoculated in a new sterile cell culture dish, with a cell density of 80% the next day advisable. Transfection of siRNA was carried out with Lipofectamine 3000 transfection reagent (Thermo Fisher Scientific, L3000015) strictly following the kit instructions. After 6 h, the medium containing the transfection reagent was discarded and replaced with a new complete medium for 48 h of culture. Real-time fluorescence-based quantitative PCR was used to verify the knockdown efficiency of siRNA, and subsequent experiments were carried out.

### Cell proliferation experiment

In this study, the proliferation ability of U343 cells after siRNA transfection was measured by CCK-8 assay. Cells in logarithmic growth phase were inoculated at a density of 3000 cells per well in 96-well plates and cultured in complete medium containing 10% fetal bovine serum. It was marked as day 0 when the cell completely adheres to the wall. CCK-8 (Keygen Biotech, Jiangsu, China) assay was used to detect cell proliferation on days 0, 1, 2, 3, or 4. The ratio of CCK-8 to DMEM-free medium was 1:10 per well. After 2 h of culture, the optical density (OD) of the well plate was measured at 490 nm using a microplate analyzer. Six repeated wells were set in each group, and the results were averaged.

### Apoptosis experiment

Cells were seeded in 6-well plates at a density of 1.0 × 105 cells/well. After transfection with siRNA for 48 h, the apoptosis experiment was performed following the instructions of Annexin V-FITC/PI Apoptosis Assay Kit (KeyGEN BioTECH, Jiangsu, China).

### RT-qPCR

Total RNA was isolated from different cell lines by lysis with TRIzol reagent (Invitrogen, USA). The reverse transcription experiment was carried out with TIANGEN Kit (QuantScript RT Kit, KR103) and operated strictly according to the instructions of the kit [17], and the resulting cDNA was used for RT-qPCR experiment. RT-qPCR was performed according to Thermo Kit instructions (PowerUp™ SYBR™ Green Master Mix, A25780). The assay was performed on 96-well plates with three duplicate wells per sample. The relative expression level of mRNA was calculated by 2^−ΔΔCt^ method, with GAPDH as the internal reference gene. Primer sequences of RT-qPCR are presented in Table 1.

**Table 1:** Primer sequences in qRT-PCR assay

	Forward	Reverse
PSMC2	5′-GGTGGCACCTACTGACATTGA-3′	5′-GTATTCCAAGGAACTCAGTCCA-3′
GAPDH	5′-CTTCATTGACCTCAACTACATGG-3′	5′-CTCGCTCCTGGAAGATGGTGAT-3′

### Western blot

RIPA buffer was used to lyse cells and extract proteins from them [18]. Bicinchoninic acid (BCA) method (P0010S; Beyotime Biotechnology) was used to determine protein concentration. Then, it was diluted at a certain proportion, washed overnight at 4 °C, and incubated with horseradish peroxide-labeled secondary antibody (diluted at a certain proportion) for 1 h. After TBST washing, it was subjected to dark room exposure and development. The name, cat. no., and working concentration of the antibodies used in the experiment are shown in Table 2

**Table 2:** Name, Cat. No. and working concentration of antibodies

Name	Manufacturer	Cat. No.	Working concentration
PSMC2	Sigma, USA	PA1-969	1:1000
Cleaved Caspase-3	Abcam, UK	ab2302	1:1000
BAX	Abcam, UK	ab32503	1:5000
BCL-2	Abcam, UK	ab32124	1:1000
GAPDH	Santa Cruz, USA	sc-47724	1:1000
Goat Anti-Mouse IgG	Bioss, Beijing, China	bs-0296G	1:3000
Goat Anti-rabbit IgG	Bioss, Beijing, China	bs-0295G	1:3000

### Statistical analysis

Western blot results were analyzed using ImageJ software. GraphPad Prism 5.0 software was used for statistical analysis. Fisher’s exact test and Student’s *t*-test were used to analyze the relative expression of PSMC2 protein in glioma and normal tissues. Two-way analysis of variance with a post hoc Bonferroni’s test was used to analyze the relative expression of PSMC2 in glioma cell lines, cell proliferation, and apoptosis. Data are presented as mean ± SD; all these experiments were conducted in triplicate. Survival analysis was performed using Kaplan-Meier survival curve. Differences with *p*-values < 0.05 were considered significant.

## Results

### PSMC2 is significantly highly expressed in gliomas

**Fig. 1:**
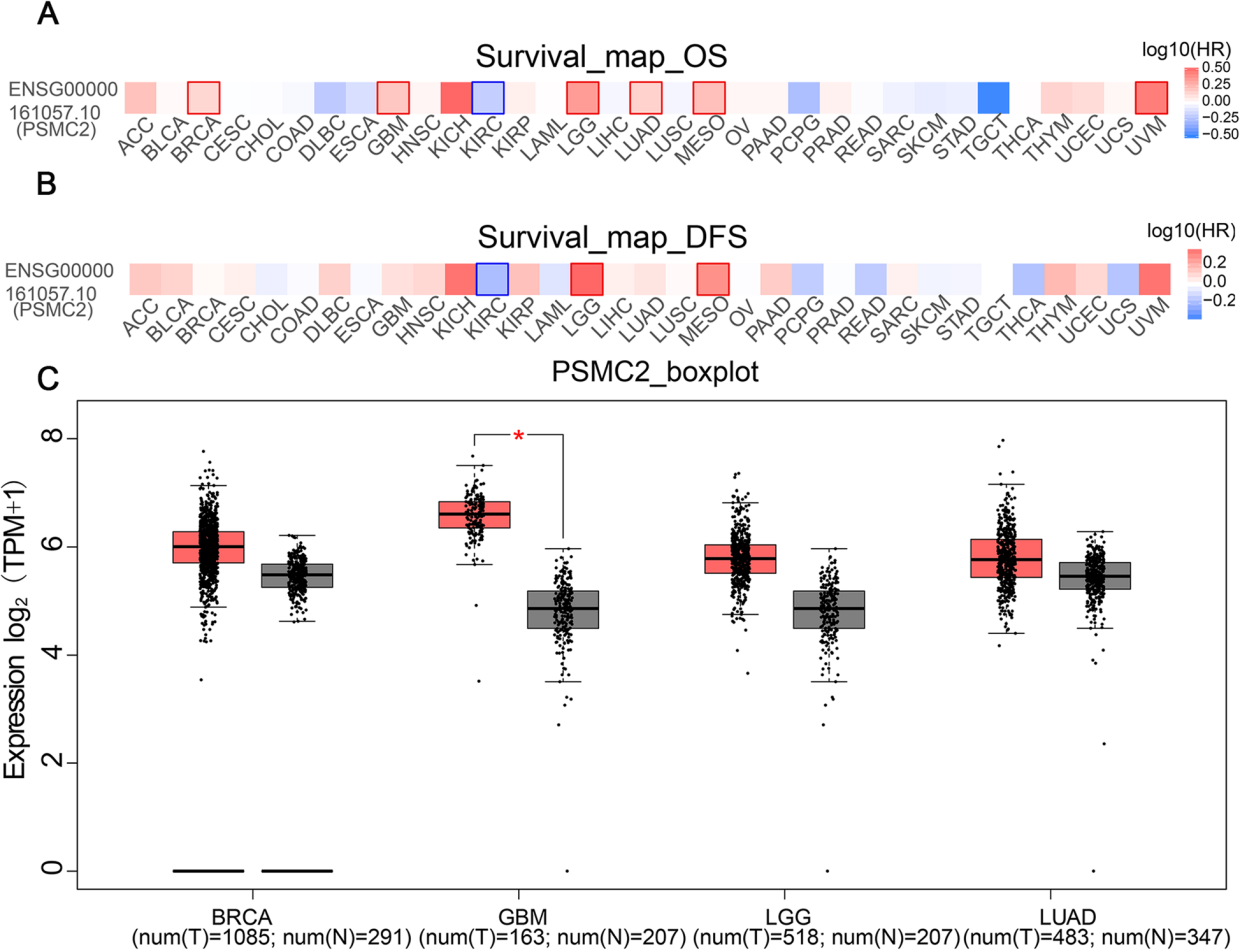
Expression of PSMC2 in tumors. **A** and **B** Overall survival (OS) and disease-free survival (DFS) correlation analysis of PSMC2 in 31 kinds of tumors. The red box represents survival risk, and the blue box represents survival protection (GEPIA). **C** Expression of PSMC2 in cancer tissues (BRCA, GBM, LGG, LUAD) and normal tissues

In order to explore the expression of PSMC2 in tumors, the expression of PSMC2 in 31 kinds of tumors was analyzed using TCGA and GETX databases (Supplementary Fig. 1). In 31 tumors, PSMC2 was significantly upregulated in BRCA, GBM, LGG, and LUAD (*p* < 0.05). GBM and LGG patients with high PSMC2 expression had a significantly worse survival (Fig. 1A and B). The measurement of PSMC2 expression in glioma tissues and control tissues revealed that PSMC2 was highly expressed in GBM (*p* < 0.001) and LGG (*p* < 0.05) tissues (Fig. 1C), and GBM was the most significant.

### General information of patients included in the study

**Fig. 2:**
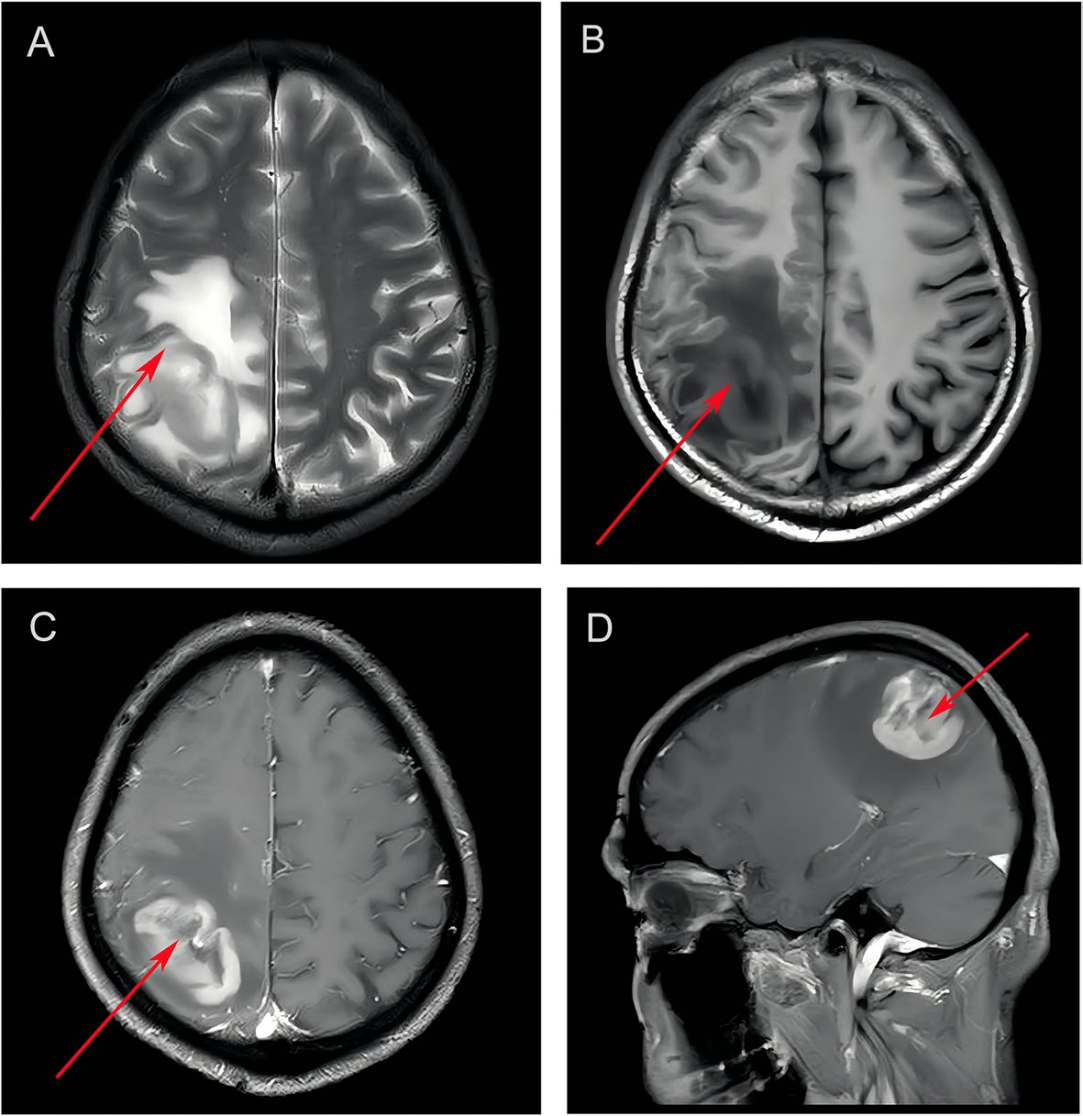
Typical brain MRI imaging results of patients with glioma. **A** Transverse plane (unenhanced MRI) T2W1. **B** Transverse plane (unenhanced MRI) T1W1. **C** Transverse plane (unenhanced MRI) enhanced T1W1. **D** Midsagittal plane (MRI) enhanced T1W1

In order to verify the expression of PSMC2 in brain gliomas, we selected a total of 60 samples in this study. Among them, there were 30 glioma samples, including 17 males and 13 females. The information is shown in Fig. 1. The age range was 22–69 years old, with an average age of 47 years. According to the 2016 World Health Organization Central Nervous System Tumor Classification [2], there were 5 cases of grade I, 8 cases of grade II, 7 cases of grade III, and 10 cases of grade IV. The diameter of the tumor was 46.21 ± 7.12 mm (Table 3). We selected the MRI images of one of the typical glioma patients. The patient was male, aged 67 years old, and clinically diagnosed as grade III glioma in the right occipito-parietal region (Fig. 2). From the T2W1 image, we can see that the patient’s right occipital-parietal region had space-occupying lesion, with iso-slightly hyperintensive signal in the center and obviously hyperintensive signal in the periphery (Fig. 2A). In the T1W1 image, the center had iso-slightly low-intensity signal, and the periphery had significant hypointense signal (Fig. 2B). T1-enhanced axial plane and T1-enhanced sagittal plane showed obvious rosette-like enhancement, with no enhancement in surrounding edema (Fig. 2C and D). There were 30 cases of negative control tissues, which were normal brain tissues from patients with craniocerebral trauma who were admitted to our hospital during the same period. There were 17 males and 13 females; the age ranged from 39 to 73 years, with an average age of 56.

**Table 3:** General information of patients included in the study

		Control group (*n* = 30)	Glioma patients group (*n* = 30)
Age (years)		39~73 (56)	22~69 (47)
Gender (Male/Female)		17/13	17/13
WHO Central Nervous System Tumor Classification	Grade I		5
	Grade II		8
	Grade III		7
	Grade IV		10
Diameter of the tumor (mm)			46.12 (7.12)

### PSMC2 increases with the increase of malignant degree of glioma

**Fig. 3:**
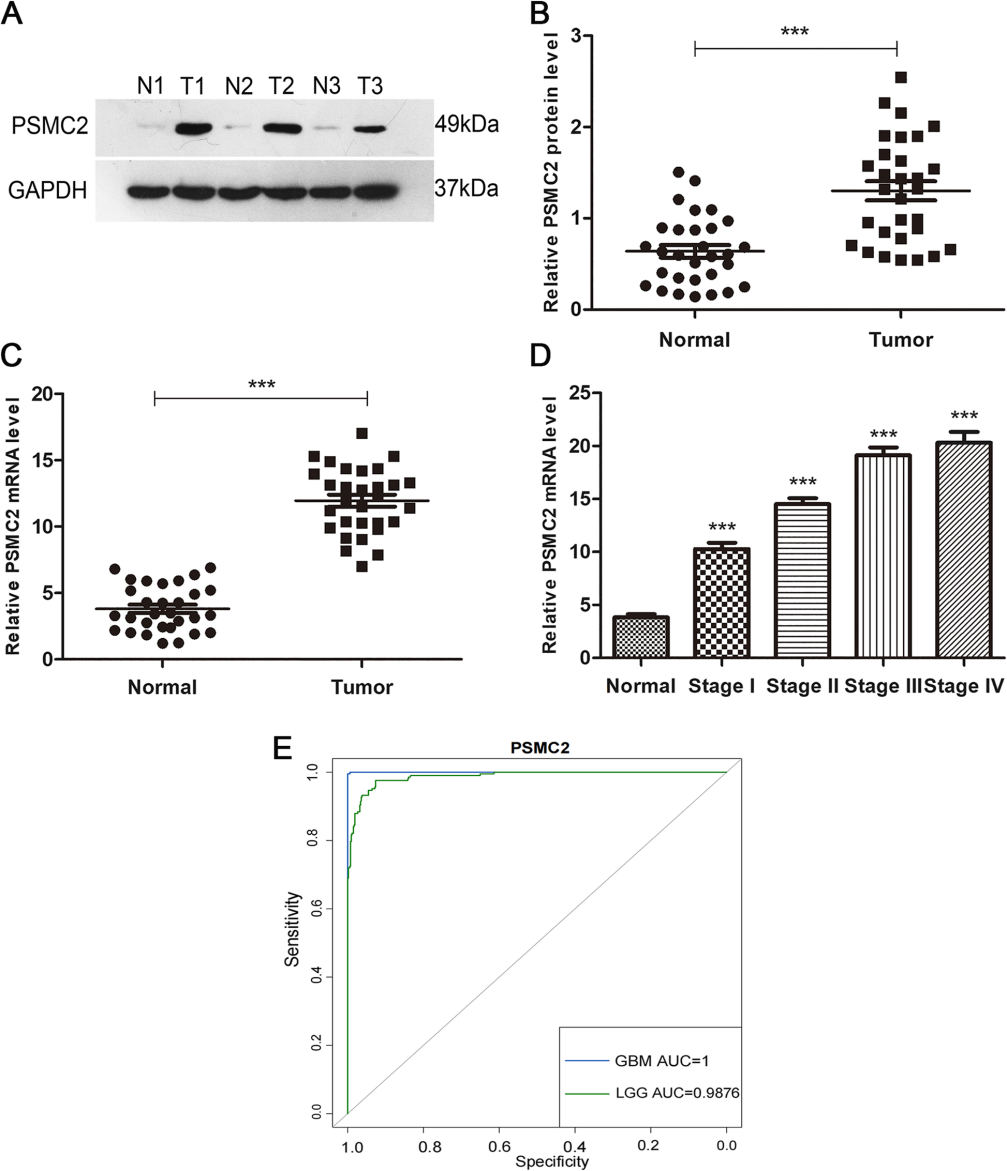
Expression of PSMC2 in glioma. **A** The protein levels of PSMC2 in three pairs of glioma tissues (T) and adjacent non-tumor tissues (N) measured by Western blot. **B** PSMC2 protein levels were quantified in glioma (*n* = 30) and normal control (*n* = 30) tissues. **C** mRNA levels of PSMC2 in 30 glioma tissues and 30 normal control tissues. **D** mRNA levels of PSMC2 in gliomas of different malignant grades, in which grade I, *n* = 5; grade II, *n* = 8; grade III, *n* = 7; and grade IV, *n* = 10. T, glioma tissue; N, normal control tissue (***p < 0.001). **E** ROC curves of differential expression of PSMC2 gene in cancer tissues (GBM, LGG) and normal tissues in the joint analysis of TCGA and GTEX databases

Western blot results showed that PSMC2 was significantly overexpressed in glioma samples (*p* < 0.05, Fig. 3A and B). RT-qPCR was performed on 30 glioma tissues and 30 normal control tissues. Compared with glioma samples, PSMC2 expression in normal brain tissues was significantly lower (N, 3.81 ± 0.93; T, 11.96 ± 1.71, Fig. 3C). According to the 2016 World Health Organization (WHO) Classification of Tumors of the Central Nervous System, gliomas were classified into I–IV grades according to malignancy. RT-qPCR showed that the higher the malignant degree of glioma, the higher the expression level of PSMC2. The average *Δ*Ct values of each group were 10.27 ± 1.33, 14.50 ± 1.60, 19.10 ± 2.03, and 20.30 ± 3.20, respectively (Fig. 3D).

Based on TCGA and GTEX databases, we constructed a receiver operating characteristic (ROC) curve model of PSMC2 in cancer tissue compared with that of normal tissue. As demonstrated in the figure, the area under the curve (AUC) of GBM is 1, and that of LGG is 0.9676, indicating that the expression of PSMC2 gene is significantly different in cancer tissues than that in in normal tissues (Fig. 3E), which is consistent with the above experimental results.

### PSMC2 expression affects the prognosis of glioma patients

**Fig. 4:**
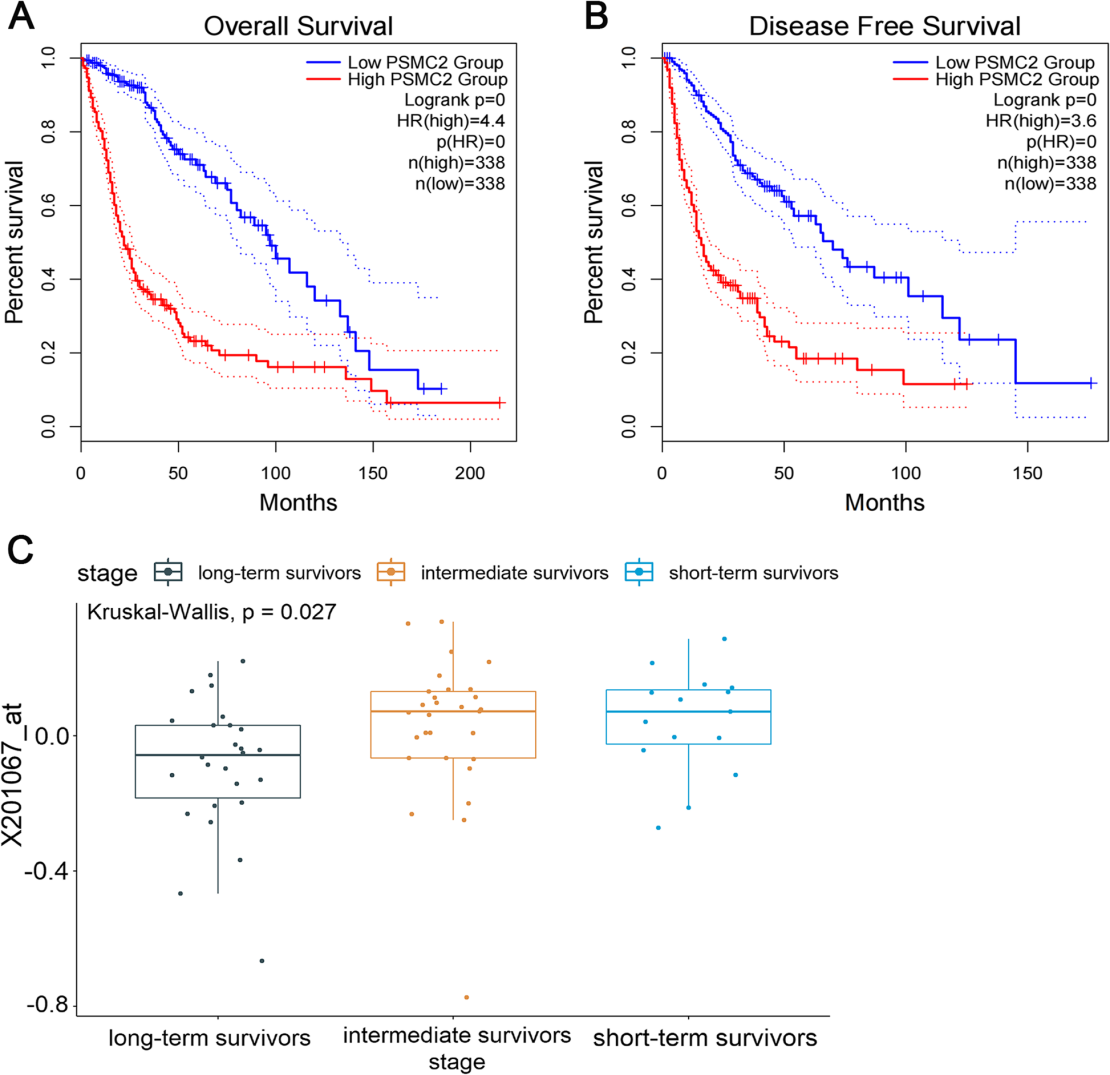
Effect of PSMC2 expression on the prognosis of glioma patients. **A** and **B** Effect of PSMC2 expression on the survival rate of glioma patients and the curves of total survival rate (**A**) and disease-free survival rate (**B**) of glioma patients with different PSMC2 levels. **C** Network boxplot of 70 German glioma patients in the GSE53733 data

In order to further explore whether PSMC2 expression affects the prognosis of glioma patients, the influence of PSMC2 expression on the overall survival rate and disease-free survival rate of glioma patients was analyzed by Kaplan-Meier survival curve based on the clinical data of patients in TCGA database. The results determined that the expression level of PSMC2 was significantly correlated with the overall survival rate and disease-free survival rate of glioma patients. The overall survival rate of patients with high PSMC2 expression was significantly worse than that of patients with low expression, with statistical difference (*p* < 0.05, Fig. 4A). Moreover, patients with high PSMC2 expression had a worse disease-free survival than those with low PSMC2 expression, and the difference was statistically significant (*p* < 0.05, Fig. 4B).

An analysis of 70 German glioma patients with malignant glioma in the GSE53733 data revealed 23 long-term survivors with a survival of more than 36 months, 16 short-term survivors with a survival of less than 12 months, and 31 survivors in between. As shown in the figure, the survival time of patients with low PSMC2 gene expression was longer, while that of patients with high PSMC2 gene expression was shorter (Fig. 4C), indicating that PSMC2 gene expression is also a factor affecting the prognosis of patients with glioma, which is consistent with the above analysis results.

### PSMC2 expression in glioma cell lines

**Fig. 5:**
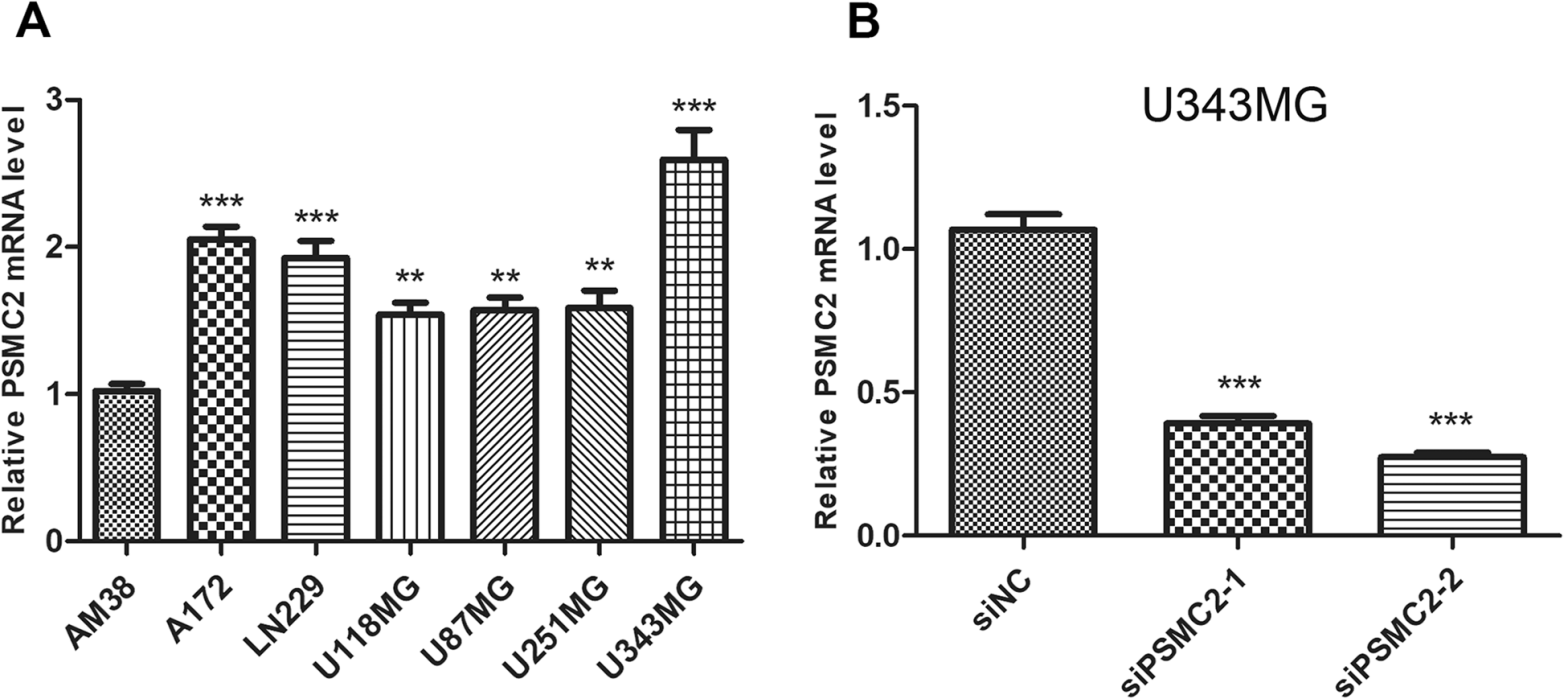
PSMC2 mRNA expression in glioma cell lines. **A** The mRNA levels of PSMC2 in seven glioma cell lines. **B** PSMC2 mRNA expression after transfection of siRNA in glioma cell line U343MG (***p < 0.001)

In order to evaluate PSMC2 expression in different glioma cell lines, we measured PSMC2 mRNA levels in 7 glioma cell lines (AM38, A172, LN229, U118MG, U87MG, U251MG, and U343MG) by RT-qPCR. Using AM38, in the cell with the lowest PSMC2 expression, as a reference, the expression of PSMC2 in the remaining six cell lines was significantly different (Fig. 5A, *p* < 0.01). U343MG, as the cell line with the highest PSMC2 expression, was selected for the subsequent PSMC2 knockdown experiment of these cells. Then, PSMC2 was knocked down by siRNA in the highly expressed cell line U343MG, and the transfection efficiency was verified by RT-qPCR (Fig. 5B). The results showed that PSMC2 levels decreased after transfection of PSMC2 siRNA, with a statistical difference between knockdown group and control group, and the knockdown efficiency of si PSMC2-2 was better. The mRNA expression levels of si NC group, si PSMC2-1 group, and si PSMC2-2 group were 1.07 ± 0.09, 0.39 ± 0.04, and 0.27 ± 0.03, respectively (*p* = 0.0008, *p* = 0.0006).

### PSMC2 promotes the proliferation of glioma cells

**Fig. 6:**
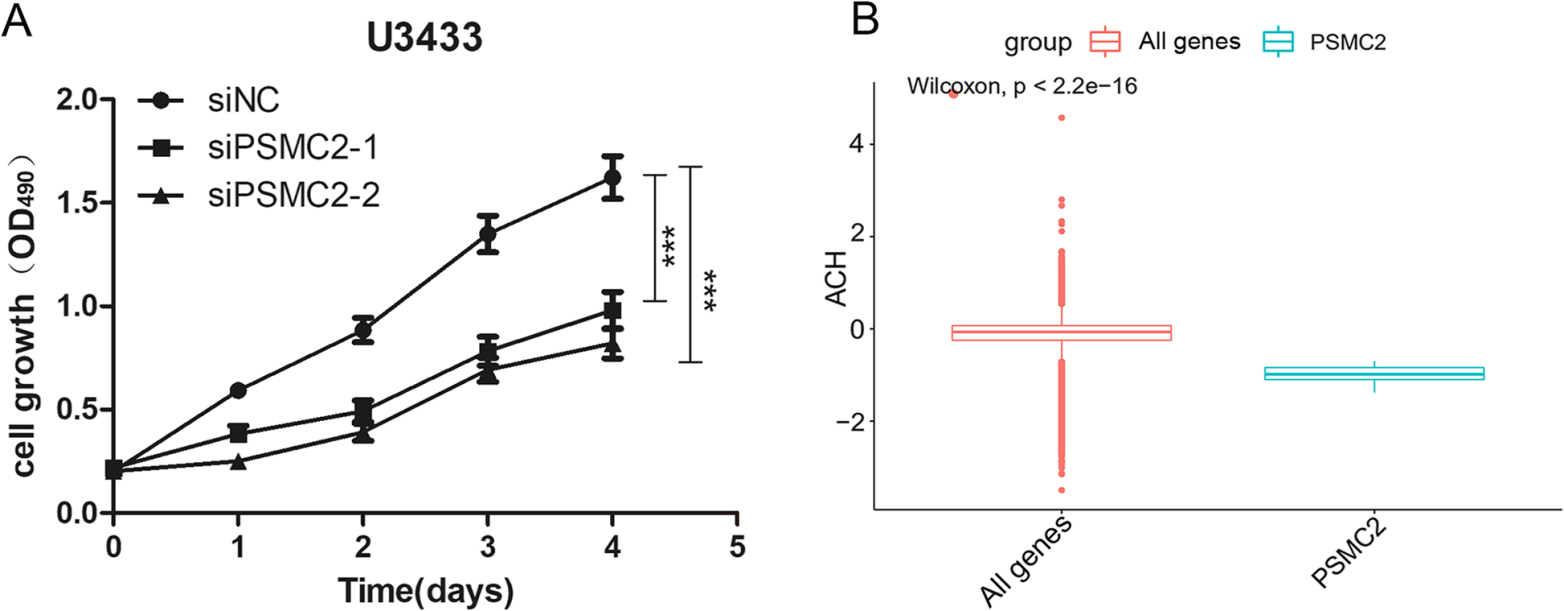
PSMC2 promotes glioma cell proliferation. **A** CCK-8 assay was used to detect the cell proliferation ability of U343MG after PSMC2 knockdown. **B** Boxplot of the PSMC2 gene-dependent score compared with that of all genes in all brain-related cell lines

To verify whether PSMC2 affects the growth of glioma cells, we performed a CCK-8 assay to evaluate the proliferation of glioma cells after PSMC2 knockdown. The results showed that knocking down PSMC2 inhibited the proliferation of U343MG cells, with a statistical difference between knockdown group and control group (Fig. 6A, *p* < 0.05). And si PSMC2-2, with more obvious knockdown effect, was more significant in inhibiting cell proliferation. These results indicate that PSMC2 can be used as a gene to promote the proliferation of glioma cells.

All the genes in all brain-related cell lines were obtained as one group and were compared with the dependent score of PSMC2 gene. Then a boxplot (Fig. 6B) was obtained. It was found that the value of PSMC2 was significantly lower than that of the other group, with a lower dependency score, indicating that cell survival was worse. PSMC2 gene was related to cell proliferation, which was consistent with the above experimental results.

### PSMC2 is associated with the apoptosis process of glioma cells

**Fig. 7:**
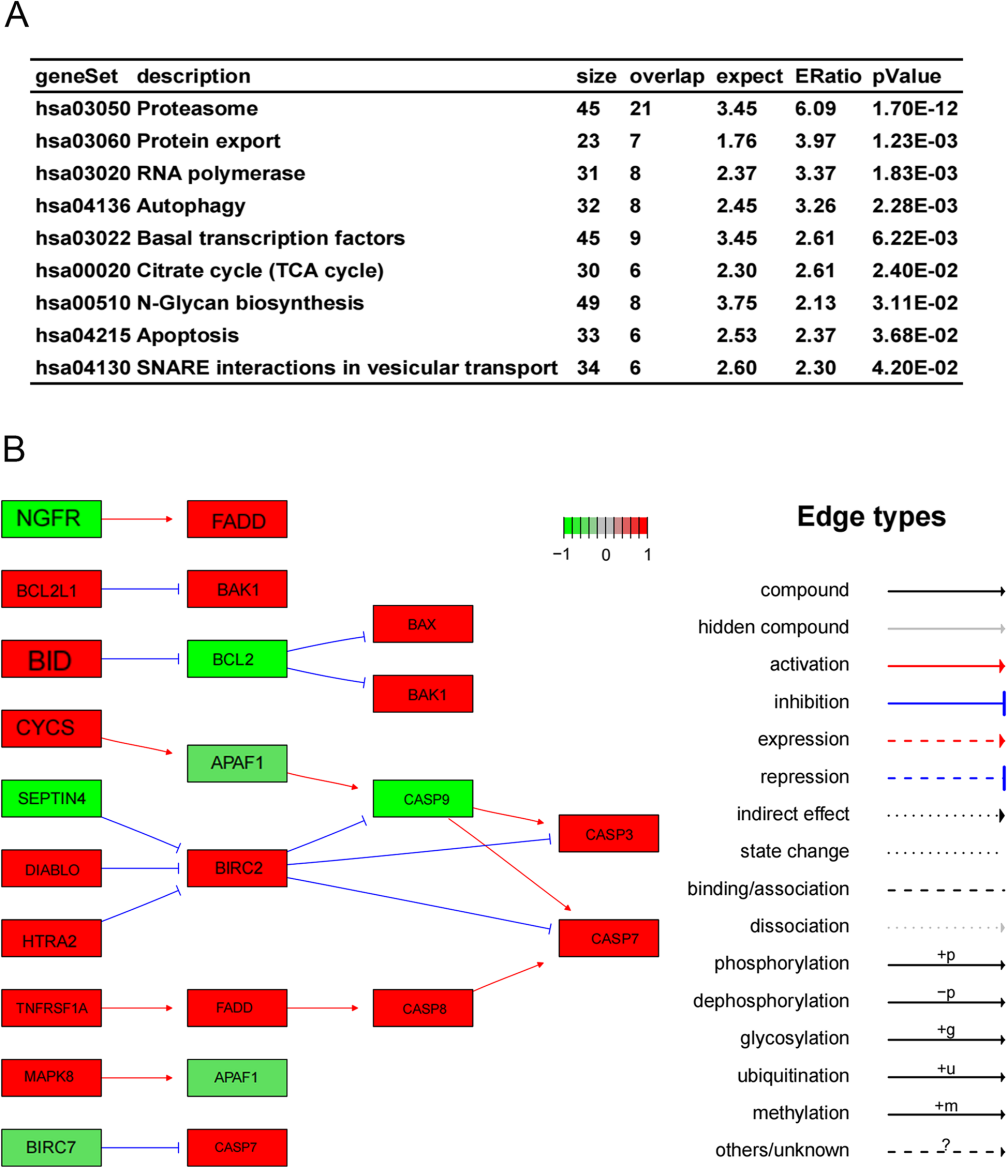
Effect of PSMC2 on apoptosis pathway of glioma cells. **A** Pathway analysis of PSMC2-influenced apoptosis process (hsa04215). **B** Pathway diagram of map of gene pathways involved in apoptosis associated with PSMC2

According to RNA-Seq data from 163 glioma patients downloaded from TCGA, we performed enrichment analysis based on the co-expression genes of PSMC2 in tumor tissues. The results showed that the co-expression genes of PSMC2 were mainly involved in the proteasome pathway and protein transport. We also found that PSMC2 affected the process of cell apoptosis (Hsa 04215; Fig. 7A). Further analysis of the pathway of PSMC2-induced apoptosis revealed that the pathway enriched 6 positively correlated genes (CYCS, CASP9, BBC3, CASP7, BAK1, and BIRC6, marked in red), and the significance was *p* = 3.68E-2 (Fig. 7B). Therefore, it can be reasonably speculated that PSMC2 may play a tumor-promoting role in gliomas through apoptosis pathway. We then carried out in vitro cell experiments to further verify its molecular mechanism.

### PSMC2 inhibits the apoptosis of glioma cells

**Fig. 8:**
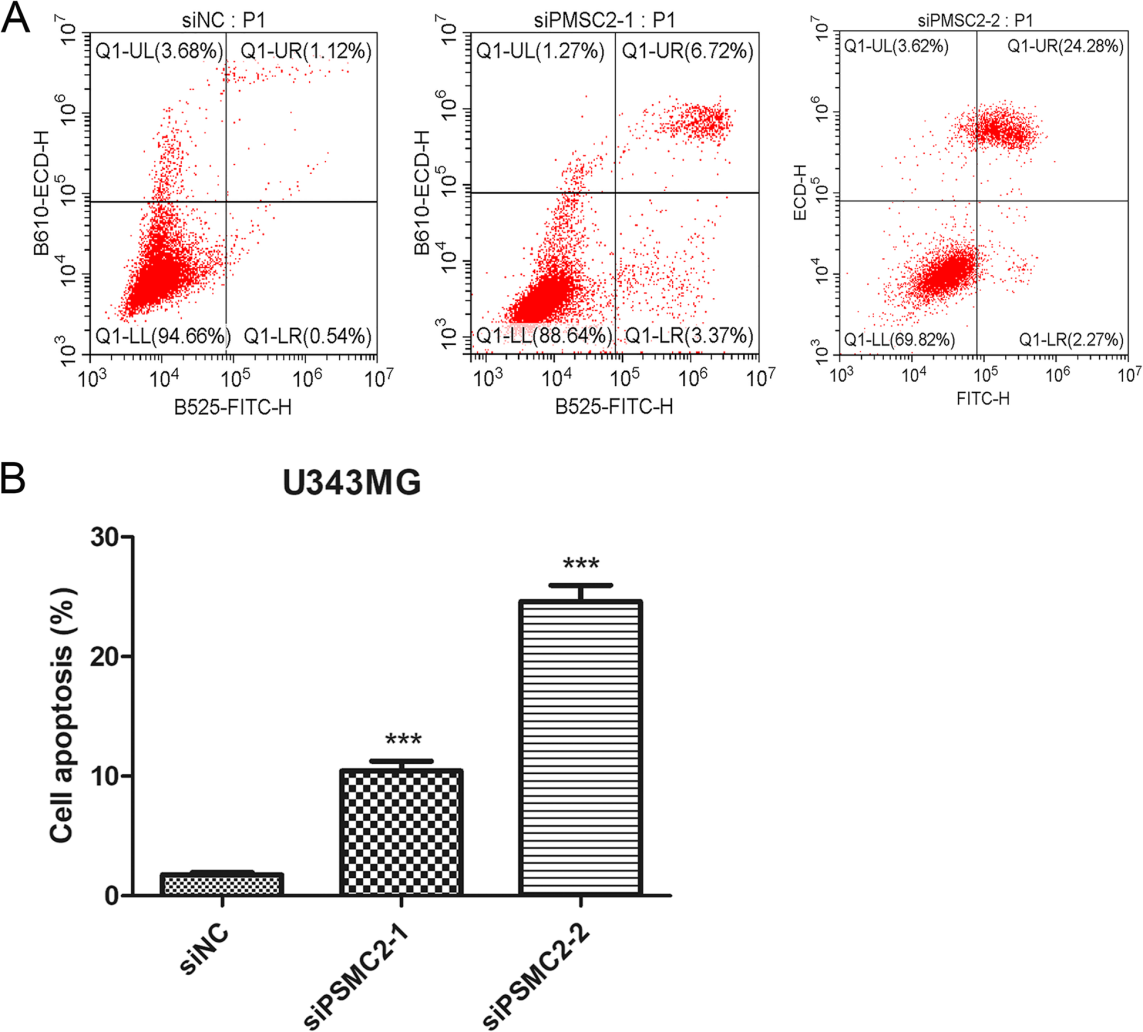
PSMC2 knockdown promotes the apoptosis of glioma cells. **A** After PSMC2 knockdown, the apoptosis level of U343MG cells was detected by flow cytometry. **B** Statistical results of three independent repeated experiments (***p < 0.001)

Annexin V-FITC/PI apoptosis assay was used to detect the effect of PSMC2 knockdown on the apoptosis ability of glioma cells. The experimental results showed that knocking down PSMC2 significantly promoted the apoptosis of U343MG cells, and si PSMC2-2, which has better knocking down efficiency, promoted the apoptosis more significantly. Apoptosis increased nearly 10 times in knockdown group compared to that in control group. The experiments were repeated three times, and it was found that the mean apoptosis rates of siNC, si PSMC2-1, and si PSMC2-2 groups were 1.77 ± 0.33, 10.45 ± 1.41, and 24.62 ± 2.35, respectively (*p* < 0.001; Fig. 8A and B). The results indicate that inhibiting the expression of PSMC2 can promote the apoptosis of glioma cells.

### PSMC2 affects the expression of apoptosis pathway-related proteins

**Fig. 9:**
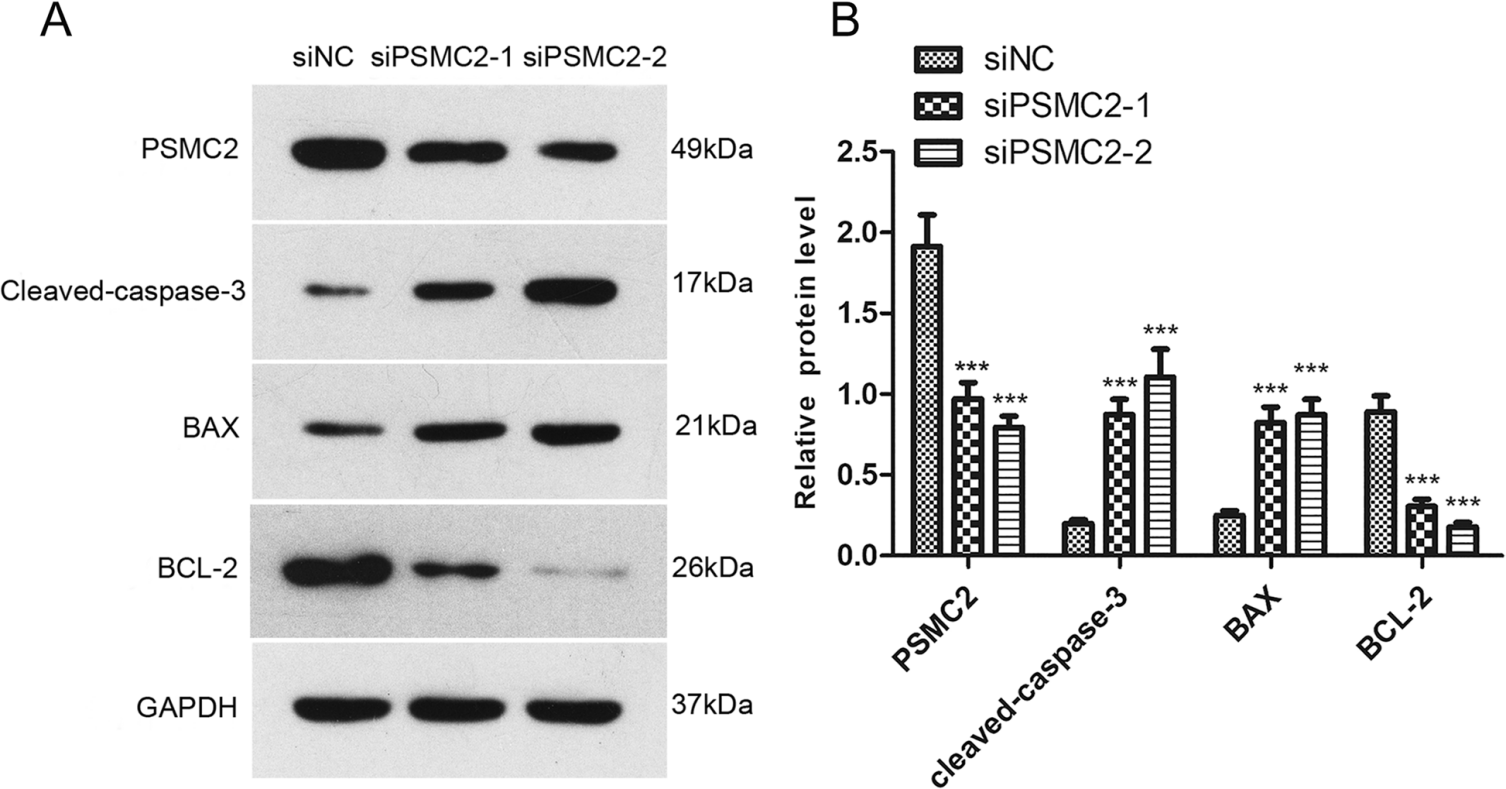
Effect of PSMC2 on apoptosis-related proteins in glioma cells. **A** Western blot of PSMC2 and apoptosis pathway-related proteins after transfection of PSMC2 siRNA into U343MG cells. **B** Statistical results of PSMC2 and apoptosis pathway-related proteins after transfection of PSMC2 siRNA into U343MG cells

To further analyze the detailed mechanism of PSMC2 affecting apoptosis process in glioma cell lines, we designed the following experiment to test whether PSMC2 expression affects the levels of apoptosis-related proteins. After transfection of PSMC2 siRNA into U343MG cell line, Western blot was applied to verify the knockdown efficiency of PSMC2 and the protein expression changes of apoptosis pathway-related proteins. The experimental results demonstrated that the expression of pro-apoptotic proteins Bax and cleaved-caspase 3 increased with the decrease of PSMC2 expression, while the expression of anti-apoptotic protein BCL-2 decreased with the decrease of PSMC2 expression (Fig. 9A and B). The above results suggest that PSMC2 may inhibit apoptosis in glioma by affecting the expression of apoptosis-related proteins.

## Discussion

Glioma, as the most important primary tumor of the central nervous system, has many subtypes leading to poor prognosis. Even with aggressive treatment strategies, radiotherapy, and chemotherapy after surgery, the problem of short survival in patients with diffuse glioma remains unsolved [19]. To effectively control the progression and spread of cancer, targeted therapy provides a new direction for personalized medicine [20].

PSMC2, part of the 19S regulatory complex of the 26S proteasome, is essential for the assembly of the 19S and 26S proteasomes [21] and guides the expansion and translocation of proteasome catalytic substrate into 20S core subunit [22, 23]. The abnormal expression of PSMC2 plays an important role in tumorigenesis and progression [24]. In this study, we used TCGA database to analyze the expression of PSMC2 in 31 kinds of tumors and found significantly upregulated PSMC2 in four tumors, especially glioma (GBM and LGG). Western blot and qRT-PCR results of clinical samples showed that PSMC2 was highly expressed in glioma tissues, and its expression level increased with the increase of tumor malignancy. Through ROC curve analysis, PSMC2 was identified as a potential diagnostic marker of glioma. The survival rate of patients with high expression of PSMC2 was significantly lower than that of patients with low expression, indicating that PSMC2 is an independent risk factor affecting the survival time of glioma patients. Therefore, we hypothesized that PSMC2 expression can be used as a biomarker to predict the prognosis of patients with glioma.

Infinite proliferation and anti-apoptosis are two important phenotypes of all malignancies. Several cancers, including glioma, can produce resistance to apoptosis. Hence, the main anticancer treatment methods are inhibition of growth and induction of cell death [25]. Inhibition of PSMC2 expression is able to inhibit the proliferation of ovarian cancer cells [14]. Similar findings have been confirmed in colorectal cancer cells, pancreatic cancer cells, and acute promyelocytic leukemia cells [15, 26]. In this study, the proliferation ability of glioma cells decreased significantly after PSMC2 knockdown and so of the apoptosis ability of glioma cells as evaluated by the Annexin V-FITC/PI apoptosis assay. It has been reported that PSMC2, as the number one gene of cancer risk caused by partial loss due to the change of copy number, is related to cell proliferation and survival [27]. Based on cell proliferation and apoptosis experiments, we speculated that high levels of PSMC2 may be conducive to the proliferation or survival of cancer cells in the tissue samples of glioma patients. According to the RNA-Seq data of 163 glioma patients downloaded from TCGA, we enriched and analyzed the co-expression genes of PSMC2 in tumor tissues. The results identified that PSMC2 affected the apoptosis pathway, which further clarified the mechanism of PSMC2 in glioma. In addition to proteolysis, the 26S proteasome is also reported to have sites for transcriptional regulation and promotion of active chromatin [28]. The 19S subunit of the 26S proteasome may also affect the diffusion of heterochromatin [29]. However, the mechanism by which PSMC2 interacts with apoptosis-related proteins during tumorigenesis has not been reported in relevant literatures. Bcl-2/Bax/cleaved caspase-3 apoptosis signal is a signaling pathway that regulates cell apoptosis and survival, which is related to a variety of diseases including nervous system diseases [30]. The Bcl-2/Bax family plays a key role in promoting or inhibiting endogenous apoptosis pathways triggered by mitochondrial dysfunction [31–33]. In our research, flow cytometry showed that the apoptosis number of U343MG cells was significantly increased after PSMC2 knockdown. Western blot revealed that the expression of pro-apoptotic proteins Bax and cleaved caspase 3 increased with the decrease of PSMC2 expression, and the expression of anti-apoptotic protein Bcl-2 decreased as PSMC2 expression reduced. Hence, PSMC2 plays a role in the apoptosis of glioma by acting on the Bcl-2/Bax/cleaved caspase-3 apoptosis signaling pathway.

Recent evidence has indicated that proteasome is essential in the proliferation and apoptosis of pancreatic cancer [16]. Twenty years ago, Theodore Pet. demonstrated that PSI, a peptide-aldehyde inhibitor of the 26S proteasome, can effectively induce apoptosis of BxPC-3 human pancreatic cancer cells [34]. According to Wang et al., rapid deletion of PSMD11 (26S proteasome non-ATPase regulatory subunit 11) can induce rapid or acute apoptosis of pancreatic cancer cells [35]. The frequency of partial genomic deletions of PSMC2 is reported to be 0.10 in 3131 cancers of multiple cancer types, indicating that cancer cells are highly dependent on the remaining PSMC2 [14]. These results further suggest that PSMC2 may be a potential target for cancer therapy. In the present study, inhibition of PSMC2 reduced cell proliferation and increased cell apoptosis, which is consistent with PSMC2 as an oncogene for osteosarcoma [13]. It suggests that the high expression of PSMC2 in glioma can inhibit apoptosis and promote cell proliferation and can be used as a therapeutic target for glioma.

## Conclusion

In this research, the RNA-Seq data of glioma patients from TCGA (The Cancer Genome Atlas) and GTEX (Genotype-Tissue Expression) databases were used. We find that PSMC2, with high expression in glioma cells, is essential for the survival of glioma cells and for the proliferation and apoptosis of glioma cells. These results suggest that PSMC2 can be used as a biomarker for the prognosis of glioma patients and a potential therapeutic target for glioma.

## Supplementary Information

**Additional file 1: Figure 1.** The mRNA expression levels of PSMC2 in 31 tumors (ACC, BLCA, BRCA, CESC, CHOL, COAD, DLBC, ESCA, GBM, HNSC, KICH, Kirc, Kirp, LAML, LGG, LIHC, Luad, LUSC, OV, PAAD, PCPG, PRAD, READ, SARC, SKCM, STAD, TGCT, THCA, THYM, UCEC, UCS) were analyzed based on TCGA database.

## Data Availability

The datasets used and/or analyzed during the present study are available from the corresponding author on reasonable request.
