# Supplementary figures and images for: PSMC2 is overexpressed in glioma and promotes proliferation and anti-apoptosis of glioma cells
Source: World J Surg Oncol. 2022 Mar 14;20:84. doi: 10.1186/s12957-022-02533-1 (PMC8922849; doi:10.1186/s12957-022-02533-1)

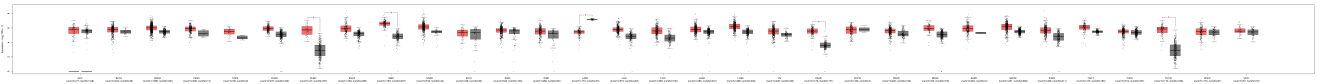

Supplement: Supplementary file 1 — Additional file 1: Figure 1. The mRNA expression levels of PSMC2 in 31 tumors (ACC, BLCA, BRCA, CESC, CHOL, COAD, DLBC, ESCA, GBM, HNSC, KICH, Kirc, Kirp, LAML, LGG, LIHC, Luad, LUSC, OV, PAAD, PCPG, PRAD, READ, SARC, SKCM, STAD, TGCT, THCA, THYM, UCEC, UCS) were analyzed based on TCGA database. [file 12957_2022_2533_MOESM1_ESM.png]
